# Utilization of custom electron bolus in head and neck radiotherapy

**DOI:** 10.1120/jacmp.v4i4.2503

**Published:** 2003-09-01

**Authors:** R. J. Kudchadker, J. A. Antolak, W. H. Morrison, P. F. Wong, K. R. Hogstrom

**Affiliations:** ^1^ Department of Radiation Physics, Department of Radiation Oncology The University of Texas, M. D. Anderson Cancer Center 1515 Holcombe Boulevard Houston Texas 77030

**Keywords:** electron bolus, electron conformal therapy

## Abstract

Conventional methods of treating superficial head and neck tumors, such as the wedge pair technique or the use of multiple electron fields of varying energies, can result in excellent tumor control. However, in some cases, these techniques irradiate healthy tissue unnecessarily and/or create hot and cold spots injunction regions, particularly in patients with complex surface contour modification or varying planning target volume (PTV) thickness. The objective of this work is to demonstrate how bolus electron conformal therapy can be used for these patients. Two patients treated using this technique are presented. The first patient was diagnosed with malignant fibrous histiocytoma involving the right ear concha and was treated with 12‐MeV electrons. The second patient was diagnosed with acinic cell carcinoma of the left parotid gland and was treated with 20‐MeV electrons after having undergone a complete parotidectomy. Each patient's bolus was designed using bolus design tools implemented in an in‐house treatment‐planning system (TPS). The bolus was fabricated using a computer‐controlled milling machine. As part of the quality assurance process to ensure proper fabrication and placement of the bolus, the patients underwent a second computed tomography (CT) scan with the bolus in place. Using that data, the final dose distribution was computed using the Philips Pinnacle^3^ TPS (Philips Medical Systems, Andover, MA). Results showed that the 90% isodose surface conformed well to the PTV and that the dose to critical structures such as cord, brain, and lung was well below tolerance limits. Both patients showed no evidence of disease six months post‐radiotherapy. In conclusion, electron bolus conformal therapy is a viable option for treating head and neck tumors, particularly patients having a variable thickness PTV or surface anatomy with surgical defects.

PACS number(s): 87.53.Kn

## I. INTRODUCTION

When radiation therapy is prescribed for patients with head and neck cancer, it is conventionally delivered via low‐energy photon beams, electron beams, or a combination of the two. Electron beams are commonly used in the treatment of lip cancer, cervical lymph nodes, and laterally located tumors, such as tumors in the parotid gland and buccal mucosa.^1^ In the past, electrons have received much less attention than *X* rays primarily due to a lack of accuracy in planning electron treatments, and the large penumbra that develops at the edge of the field. Further, matching electron fields of differing energy can be problematic due to not having matched penumbras, leading to dose inhomogeneity (hot and cold spots) in the abutment regions. However, the advantage of electron beams in radiation therapy is that their finite range in tissue delivers the desired dose to the target volume while sparing distal organs at risk. This property alone, gives electron beams the distinct advantage in treating tumors located at limited depth.

Wedge‐pair arrangements using photon beams spare the opposite side of the oral cavity in unilateralized lesions, such as those of the buccal mucosa or parotid gland. However, the volume of tissue irradiated to full dose is excessive, increasing the probability for future complications. In addition, for postoperative irradiation of a radically dissected neck, anterior, and posterior photon beams cannot be used because their use would lead to over‐irradiation of the mucous membranes of the mouth and throat. With electron beams, unilateral irradiation of the buccal mucosa, or parotid gland, or radical neck dissection can be achieved with minimal dose to deep structures. Also, after surgical procedures where there is a high risk of recurrence, electron beams are the most appropriate modality because treatment is difficult to achieve using photon beams.^1^

It is well known that irradiation of patient surfaces that have surgical defects with electron beams will lead to significant dose heterogeneity in underlying tissue.^2^ ^,^ ^3^ This dose heterogeneity can lead to planning target volume (PTV) underdose and unwanted side effects and complications. A common technique to solving the problem of dose inhomogeneity when treating head and neck tumors with electrons is to use bolus as a missing tissue compensator. The objective of this technique is to make the patient surface anatomy as much like a water phantom as possible, which would result in a more homogeneous dose distribution.^3^ ^–^ ^6^ In addition to the irregular patient surface commonly seen in head and neck patients, the PTV thickness is not usually uniform and may sometimes vary from less than 1 cm to greater than 5 cm. If a single electron beam (with or without a uniform thickness bolus on the patient's surface) were used to treat such patients, critical underlying tissue would be unnecessarily irradiated. Therefore, such cases might employ multiple electron beams of different energies used simultaneously to irradiate different sections of a target with variable depth. This technique, however, is associated with junction problems resulting from abutting adjacent treatment fields that could lead to hot and cold spots.^1^ ^,^ ^7^ Also, using a greater number of treatment fields results in a greater conformity of the 90% dose surface to the PTV, at the expense of multiple entries in the treatment room to setup individual fields. A clinical solution to irregular patient surface and nonuniform PTV thickness is the use of conformal electron radiation therapy using variable thickness wax bolus, which is customized for the individual patient. The custom‐designed electron bolus allows shaping of the 90% dose surface to the PTV while simultaneously excluding as much surrounding healthy tissue as possible.

Intensity modulated *x*‐ray therapy (IMXT) is used often in the management of head and neck cancer, as its ability to conform the dose to the PTV limits the dose to critical structures or pre‐irradiated areas. A typical IMXT treatment plan consists of multiple *x*‐ray beams incident on the patient at multiple angles and has been proven highly useful in head and neck radiation therapy. Clearly, electron conformal therapy cannot treat mid‐line tumors; however, due to the finite range of electrons in tissue, bolus electron conformal therapy can offer some advantages over IMXT in pediatric patients or in adults where the PTV is within 6 cm of the surface. With bolus electron conformal therapy only one beam is used, usually reducing the amount of unnecessary low dose to healthy tissue surrounding the tumor receives.

The use of custom‐shaped electron bolus at M. D. Anderson Cancer Center is not a new technique. The technique was demonstrated at our clinic by Low *et al*. in the treatment of paraspinal muscles^8^ and by Perkins *et al*. in post‐mastectomy irradiation.^9^ This manuscript describes the treatment planning and delivery of a custom three‐dimensional (3D) electron bolus for electron beam radiation therapy for tumors located in the head and neck region in order to demonstrate the usefulness of this technique. Two patients are presented: one patient was diagnosed with malignant fibrous histiocytoma involving the right ear concha and the other with acinic cell carcinoma of the left parotid gland.

Radiation therapy using custom 3D electron bolus for head and neck tumors is more challenging and involved than other tumor sites. Design of electron bolus for head and neck tumors is unique in that the PTV has a more complex shape, the critical structures and their relationship to the PTV are different, and the patient surface is more irregular. In this manuscript we have described a new commercially available bolus fabrication process. For the bolus quality assurance (QA) and final treatment plan, for the first time a commercial 3D treatment planning system (TPS) Pinnacle^3^ (Philips Medical Systems, Andover, MA) was used.

## II. MATERIALS AND METHODS

The treatment‐planning and delivery methods used for bolus electron conformal therapy for our two patients are fully integrated and are described as a series of specific steps. These steps are: (i) acquisition of a 3D model of the patient using computed tomography (CT), (ii) development of a treatment plan using a 3D treatment‐planning system equipped with proper tools, (iii) physician approval of the dose plan, (iv) fabrication of the electron bolus and other patient‐specific treatment hardware, (v) QA to assess the quality of the electron bolus and plan, and (vi) patient setup and delivery.

### A. Computed tomography data acquisition

First, each patient was placed in the treatment position on the simulator table. The patient's head and neck were then immobilized using a perforated thermal plastic immobilization system (Aquaplast, Wycoff, NJ) immobilization system while the patient's body was supported with a Vac‐Fix vacuum bag (Soule Corporation, Tampa, FL). After 24 h, each patient underwent a CT scan, with the preliminary treatment field marked on the patient using radio‐opaque wires. Multiple CT slices (3 mm slice width and 3 mm separation) were acquired and transferred to the 3D TPS Pinnacle^3^. Once the CT images of the patient were loaded into the TPS, the radiation oncologist outlined the PTV, i.e., the target volume that should receive 90% of the given dose (given dose is the maximum central‐axis dose delivered in a water phantom at the same source‐to‐surface distance (SSD) and with the smallest rectangular field that encompasses the irregular field used on the patient).^10^ The patient's skin surface and associated critical structures were also outlined. The CT data and contours were then exported in the Radiation Therapy Oncology Group format to the in‐house TPS COPPERPlan.^11^ ^,^ ^12^

### B. Initial plan for bolus design

The beam parameters were chosen once all the CT data and contours had been imported into COPPERPlan. The gantry angle was chosen so that, from the beam's perspective, the distal side of the PTV was approximately perpendicular to the beam direction. The SSD was set to approximately 105 cm to allow for the electron bolus to be placed on the patient without collision with the applicator. Based on the maximum depth of the PTV, the electron beam of least energy, such that its $R_{90}$ (central‐axis depth of 90% dose) was greater than the maximum depth of the PTV, was chosen. The field size was then determined in beam's eye view (BEV); an irregular field was drawn with a margin of 1 to 2 cm around the PTV

Bolus design operators, which assist the treatment planner in conforming the 90% isodose surface to the distal surface of the PTV and reduce dose to critical structures, have been developed by Low *et al*. and have been implemented into COPPERPlan.^13^ Three classes of bolus operators were used: a creation operator created the initial thickness of the bolus based on the physical depth of the target volume, modification operators modified the bolus shape, and an extension operator extended the bolus outside of the field to account for beam penumbra. The bolus shape and resulting dose distribution were then computed in COPPERPlan, the latter using a 3D implementation of the electron pencil‐beam algorithm to calculate dose.^11^ ^,^ ^14^ The 3D planning system models the bolus as a combination of two surfaces: the distal surface and the proximal surface. The distal surface is the surface that is in contact with the patient and is obtained by ray tracing from the electron source to the patient's surface. The proximal surface lies closer to the source and is computed using the operators mentioned above. Treatment plans with electron bolus can be evaluated by viewing isodose contours in transverse, sagittal, and coronal planes as well as dose‐volume histograms (DVHs). After a satisfactory treatment plan was obtained it was approved by the physician.

### C. Bolus fabrication

Using the approved treatment plan, the electron bolus was fabricated with a computer‐driven milling machine. The bolus was machined from modeling wax, which has a density of approximately 0.92 gm/cm^3^.^8^ ^,^ ^15^ The surface coordinates of the proximal and distal surfaces obtained from the TPS were read into the computer‐aided design‐computer aided manufacturing (CAD‐CAM) software (EdgeCAM; Pathtrace Systems Inc., Southfield, MI). Using this input, EdgeCAM generated the necessary surface models and then created the machine toolpath data. This data was then transferred using the Servo software (Servo Products Company, Pasadena, CA) to the Bridgeport milling machine (Bridgeport Machines Inc., Bridgeport, CT) for the production of the final machined bolus. It took about 4 h to mill the proximal and distal surfaces of each bolus.

Electron bolus could also be milled by a third party, as are compensators. We recently signed a contract with .Decimal, a division of Southeastern Radiation Products Inc., (Sanford, FL) to fabricate bolus for us. A file containing the surface coordinates of the proximal and distal bolus is emailed to .Decimal, and within 24 h, the milled bolus is shipped to us.

### D. Bolus quality assurance and final plan

To ensure proper fabrication and placement of the electron bolus, the patient underwent another CT with the bolus in place. The second CT acts as a QA tool to ensure that the bolus is properly manufactured and to provide the CT data that is used for the final treatment plan.^16^ ^,^ ^17^ Fiducial marks were made on the bolus to assist with the alignment of the bolus on the patient's surface. BB's were placed along the fiducial marks as well. Care was taken while placing the bolus on the patient so that minimal air‐gaps were introduced between the bolus and the patient. Air‐gaps, if present, could alter the intended dose distribution. Once the CT scan was completed, the data was transferred to the 3D TPS Pinnacle^3^, and the dose distribution was computed. This dose distribution was visually compared with the dose distribution of the bolus design plan originally calculated using COPPERPlan to ensure agreement between the two. The tolerance we have set for our bolus electron conformal therapy treatment plans, is 90% of the isodose surface must agree to within ±2 mm and the dose within the 90% isodose surface must agree to within ±3%. The radiation oncologist was asked to approve and sign the final dose plan, which was placed into the patient's treatment chart. If it turns out that the difference marginally exceeds our criteria, the plan may still be clinically acceptable as long as the radiation oncologist is satisfied with the plan.

### E. Daily treatment

The patients were treated with the electron bolus in place, using a single field. Accurate and reproducible positioning of the bolus on the patient on a daily basis is critical. The bolus was placed on the patient and the fidicual marks on the bolus were aligned with the skin marks using the positioning lasers. The bolus was immobilized using the lips on the mask or with masking tape. The bolus was carefully docked with the skin surface and was assured to be in close contact with the skin. Care was taken to place the bolus correctly on each day of treatment. If bolus fit is suspect at any time during the treatment process, then re‐CT is required to verify its acceptability, although this has not been required for any of the patients treated thus far.

## III. RESULTS AND DISCUSSION

### A. Patient 1 (ear concha case)

The patient is a 52–year‐old man who was diagnosed with malignant fibrous histiocytoma involving the right ear concha. Physical examination in the area of the right ear showed postsurgical changes from previous surgeries with partial loss of his right ear and the presence of a split‐thickness graft in the preauricular area. A 4.5–cm×4.5–cm splint‐thickness skin graft was present in the right preauricular region. Previous graftings were present in the right concha. The external auditory canal showed no suspicious lesions. There was a 1‐cm area of healing tissue in the superior portion of the concha, which had no recurrent disease. The postauricular region had no recurrence. There were no palpable neck nodes or parotid nodes present.

**Figure 1:**
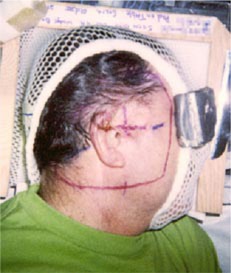
(Color) View of Patient 1 immobilization. A thermal plastic patient immobilization system (Aquaplast, Wycoff, NJ) is used to immobilize the patient's head and neck.

**Figure 2:**
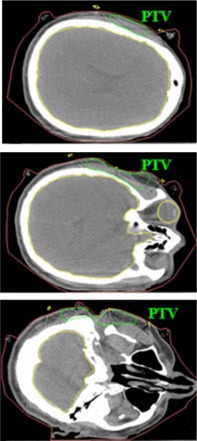
(Color) Transverse CT slices of Patient 1 showing the location, depth, and shape of the parotid PTV at various levels.

**Figure 3:**
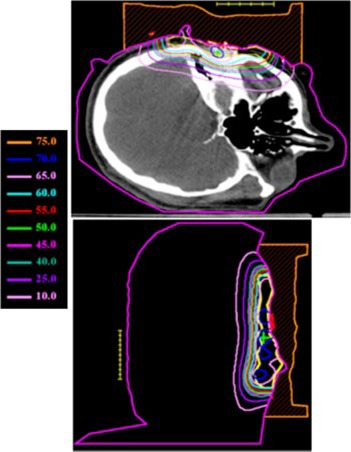
(Color) Transverse and sagittal isodose distribution (Gy) for initial bolus design plan for Patient 1. A dose of 60 Gy was prescribed to the 90% of the given dose using 12 MeV electrons. The PTV is indicated with the yellow contour.

The treatment plan was to irradiate all areas that had been previously surgically violated and resected using an appositional electron field of 12 MeV. A dose of 60 Gy was prescribed to the 90% isodose surface, which was to conform as closely as possible to the distal surface of the PTV Initially, a 2‐cm tapered bolus placed in the upper part of the field to decrease the dose to the underlying brain tissue was considered. However, the physician wanted greater dose conformity, which could be achieved using a custom 3D electron bolus. The custom bolus would provide better dose coverage of the PTV while minimizing dose to surrounding critical structures. Water was used to fill in the external auditory canal, eliminating air so that a more homogeneous dose distribution could be achieved. Figure 1 shows the immobilized patient and surface anatomy relative to the field border. The patient CT data was transferred to the COPPERPlan TPS and the PTV, skin surface, and critical structures (brain, right orbit, spinal cord) were contoured. Figure 2 shows the varying PTV thickness and skin surface‐gradient on three axial CT slices. The electron beam was for a gantry angle of 0 degrees, i.e., from top to bottom in Fig. 3 transverse plane view. The rectangular field circumbscribing the irregular field was 15 cm×15 cm, and the patient SSD was 105 cm. The calculated dose under the designed bolus is shown in Fig. 3 in a transverse and sagittal plane. The 60 Gy contour (turquoise) represents the 90% isodose contour, and it conforms well to the distal PTV surface. The dose in the PTV ranges from 60–70 Gy with the exception of a small volume exceeding 70 Gy, which is due to the irregular surface of bolus. The dose inhomogeneity could be reduced using intensity modulation if it were available.^18^

**Figure 4:**
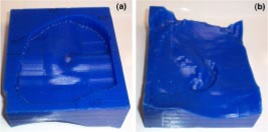
(Color) (a) Custom electron bolus proximal surface used to treat Patient 1. The proximal side is designed to modulate the penetration of the electron beam to match the PTV. (b) Custom electron bolus distal surface used to treat Patient 1. The distal side is designed to match the patient skin surface.

**Figure 5:**
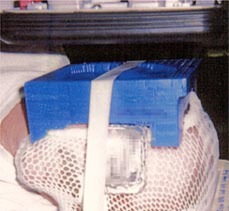
(Color) The custom 3D electron bolus in treatment position for Patient 1.

**Figure 6:**
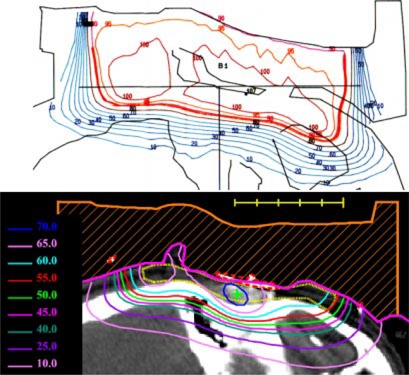
(Color) Comparison of isodose distribution with bolus in place QA plan and bolus design plan for Patient 1 (Note: Prescription 60 Gy at 90% i.e. 90% 2D plan equals 60 Gy 3D plan).

**Figure 7:**
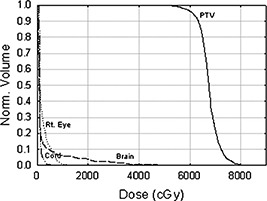
Dose volume histograms for the customized electron bolus plan for Patient 1. The histograms show that the electron bolus treatment plan covers the target volume well while minimizing dose to the critical organs.

The manufactured bolus proximal and distal surfaces are shown in Figs. 4(a) and 4(b), respectively, and Fig. 5 shows the bolus in treatment position. Our QA process required that a second CT scan be performed with the bolus placed on the phantom to obtain the final dose plan. Figure 6 shows a comparison between the isodose distribution on the central axial slice with the custom bolus in place and the dose coverage for the bolus design plan. The 90% isodose line in the design plan matches well with the 60 Gy prescription line in the final bolus QA plan. Figure 7 shows the DVH for the PTV and critical organs. The small volume (approximately 4%) under the 60 Gy‐prescribed dose is not a result of the bolus design, but rather a result of the field edge being close to the interior border of the PTV as seen in Fig. 3. Had there been a clinical decision to increase the field size, this would not have existed. The increase in dose above 66 Gy (given dose) is due to scatter from the irregular surface, as previously discussed. Dose to the right eye is less than 12 Gy, dose to the spinal cord is less than 10 Gy, and dose to the brain is less than 40 Gy, with only about 5% above 20 Gy.

Due to the introduction of the electron bolus, the skin dose increases. Physical examination during treatment revealed brisk erythema of the treated field. However, there was no apparent moist desquamation. Six months after radiation therapy, the patient had no evidence of recurrence.

### B. Patient 2 (parotid gland case)

**Figure 8:**
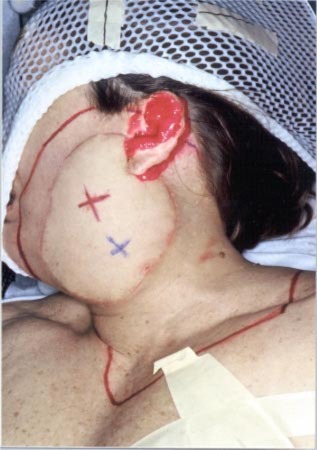
(Color) View of Patient 2 immobilization. A thermal plastic patient immobilization system (Aquaplast, Wycoff, NJ) is used to immobilize the patient's head and neck.

**Figure 9:**
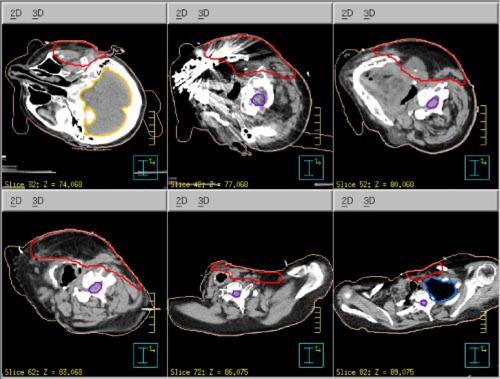
(Color) Transverse CT slices of Patient 2 showing the surface anatomical defect and the location, depth, and shape of the parotid PTV at various levels.

The patient is a 61‐year‐old woman. She was diagnosed with acinic cell carcinoma of the left parotid gland and had a complete parotidectomy with lymph node dissection with 0/14 positive nodes. She presented for postoperative radiation therapy. In addition to the surgical defect and large gradients on the surface skin contour shown in Fig. 8, the PTV thickness varied from <1 cm to >5 cm, shown in Fig. 9. Hence, instead of using segmented electron fields (two abutted fields of different energies), it was decided that conformal electron radiation therapy using custom electron bolus would be used as the treatment technique for this patient. The treatment plan was to the postoperative site using a left open lateral technique. To show the advantages of the use of custom electron bolus in this particular instance instead of the conventional treatment technique using two patched fields, a conventional treatment plan was also created with electron energies of 12 MeV and 20 MeV.

**Figure 10:**
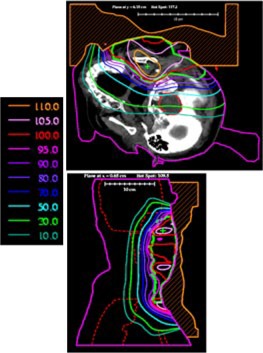
(Color) Transverse and sagittal isodose distribution (%) for initial bolus design plan for Patient 2. The maximum depth of the PTV is such that 20 MeV electrons were required for the bolus electron conformal treatment plan. The PTV is indicated with the green contour.

Then, to achieve a more homogeneous dose distribution prior to the CT scan, moldable TX‐150 was placed inside the patient's auditory canal and around the external ear to eliminate air pockets between the patient and bolus (cf Fig. 8). One field was used combining a 20‐MeV electron beam and 6‐MV *x*‐ray beam with a 4:1 mix of given dose to 54 Gy in 27 fractions. The dose distribution from the electron contribution along an axial and sagittal slice is shown in Fig. 10. Following a field reduction, the dose was taken to 60 Gy. Due to the higher energy electron beam, the surface dose is higher, leading to more intense skin reactions. Also, due to the introduction of the electron bolus, the skin dose increases. The intensity of the skin reactions at the higher energies makes it necessary to combine electrons with megavoltage photons.^1^ Hence, the 6–MV *x*‐ray beam was introduced into the treatment for one fraction each week to minimize the effect of erythema and moist desquamation.

**Figure 11:**
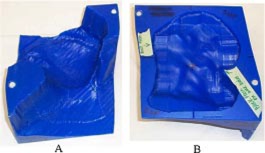
(Color) (a) Custom electron bolus distal surface used to treat Patient 2. The distal side is designed to match the patient surface. (b) Custom electron bolus proximal surface used to treat Patient 2. The proximal side is designed to modulate the penetration of the electron beam to match the PTV.

**Figure 12:**
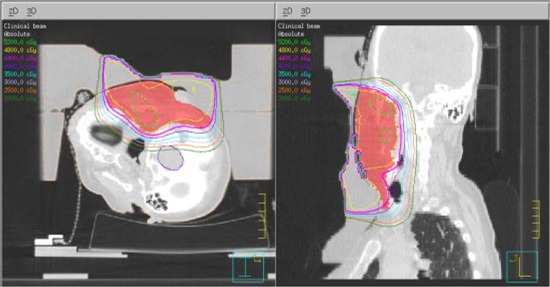
(Color) Isodose distribution with bolus in place QA plan for Patient 2.

**Figure 13:**
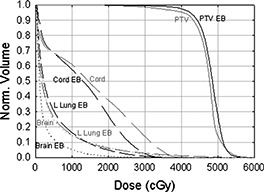
Dose‐volume histograms for the PTV, spinal cord, left lung, and brain for the custom bolus treatment plan (black lines) and a two‐field plan with a 20 MeV electrons superiorly and 12‐MeV electrons inferiorly (gray lines).

The electron bolus was then fabricated; the proximal and distal surfaces are shown in Figs. 11(a) and 11(b), respectively. The electron bolus was used throughout the treatment for the electron component of the treatment. The isodose distribution in representative transverse and sagittal planes is shown in Fig. 12. The dose in the PTV ranges from approximately 40–55 Gy with the exception of a small volume under 40 Gy, which is due to the slightly smaller margin applied to generate the electron cutout. A slight increase in the margin would have ensured better coverage of the PTV. A DVH comparison of the conventional patched electron field technique and the custom electron bolus treatment technique is shown in Fig. 13. As seen in the figure, the PTV coverage is better, and the cord and brain doses are substantially lower in the electron bolus treatment plan, whereas the left lung dose is comparable with both treatment plans.

Physical examination during treatment revealed brisk erythema overlying the treatment fields with no adenopathy or masses palpated within the operative bed. Examination of the oral cavity and buccal cavity during treatment also revealed slight patchy mucositis in the superior buccal cavity. The patients skin recovered approximately two weeks after radiation therapy. Six months after radiation therapy, the patient presented with no evidence of disease and no complaints related to mucositis or skin dermatitis.

## IV. CONCLUSIONS

The present work illustrates how easily bolus electron conformal therapy can be planned to treat superficial head and neck cancers. The treatment for the two cases, histiocytoma of the right ear concha and acinic cell carcinoma of the left parotid gland, showed significant conformity of the 90% dose surface to the PTV with little dose to critical structures surrounding the PTV. In comparison, conventional techniques such as the wedge‐pair technique or multiple electron fields of varying energy lead to more healthy tissue being unnecessarily irradiated or the introduction of hot and cold spots injunction regions.

Bolus electron conformal therapy offers a useful method for achieving electron conformal therapy in head and neck cancer, especially in cases where the patient's surface anatomy has surgical defects and where the PTV is near the patient's surface and has a variable thickness. The increased dose due to bolus is easily managed.

Bolus electron conformal therapy could be widely used by the radiotherapy community if TPS manufacturers would include the bolus design technology of Low *et al*.^13^ into their TPS. On the other hand, the bolus fabrication issue has not been so bad. As most clinics could not support this technology, we have identified a third party, .Decimal, which can receive, mill, and overnight mail a machined bolus to the clinic within 24 h

Future work should include demonstrating the utility of bolus electron conformal therapy in addition to intensity modulated *x*‐ray therapy in patients with head and neck cancer for tumors located near the surface.
